# Supplementary material for: Improving Variational Autoencoders for New Physics Detection at the LHC With Normalizing Flows
Source: Front Big Data. 2022 Feb 28;5:803685. doi: 10.3389/fdata.2022.803685 (PMC8919050; doi:10.3389/fdata.2022.803685)
Supplement: Supplementary file 1 [file Data_Sheet_1.PDF]

Supplementary Material

1 MODEL VARIANCE

In general, deep learning models are bound to have a variance in the results arising from different trainings with different random seeds for the stochastic gradient descent optimization method. To ensure that does not affect our inferences based on the final anomaly identification results, we performed five separate trainings with different random seeds. Table S1 summarizes the median anomaly identification performance across all channels with the added variance across these five separate trainings, for the Conv-VAE model, the baseline GCN-VAE, and the GCN-VAE with the addition of the various normalizing flow models. Compared to the observed uncertainties, the improvements discussed in the paper are statistically significant.

Table S1. Anomaly detection performance across all channels, combined based on median scores along with the variance over multiple trainings.

Model	AUC	$\epsilon_S(\epsilon_B = 10^{-2})$	$\epsilon_S(\epsilon_B = 10^{-3})$	$\epsilon_S(\epsilon_B = 10^{-4})$
Conv-VAE	$75.6\% \pm 0.5\%$	$1.7\% \pm 0.6\%$	$0.26\% \pm 0.04\%$	$0.13\% \pm 0.07\%$
GCN-VAE	$76.8\% \pm 0.2\%$	$55.7\% \pm 1.1\%$	$16.2\% \pm 0.6\%$	$7.1\% \pm 0.1\%$
**GCN-VAE_HouseholderSNF**	$\mathbf{86.1\% \pm 0.4\%}$	$\mathbf{69.6\% \pm 1.9\%}$	$\mathbf{34.2\% \pm 0.9\%}$	$\mathbf{8.2\% \pm 0.5\%}$
GCN-VAE_OrthogonalSNF	$82.4\% \pm 0.6\%$	$65.0\% \pm 1.4\%$	$16.1\% \pm 1.4\%$	$7.6\% \pm 0.1\%$
GCN-VAE_NSFAR	$82.1\% \pm 0.4\%$	$64.1\% \pm 1.8\%$	$15.6\% \pm 1.0\%$	$5.1\% \pm 0.3\%$
GCN-VAE_PlanarFlow	$80.0\% \pm 0.7\%$	$61.1\% \pm 1.4\%$	$16.2\% \pm 1.2\%$	$7.1\% \pm 0.1\%$
GCN-VAE_ConvolutionalFlow	$79.2\% \pm 0.2\%$	$62.7\% \pm 1.4\%$	$16.0\% \pm 1.4\%$	$6.0\% \pm 0.2\%$
GCN-VAE_TriangularSNF	$75.9\% \pm 0.5\%$	$64.7\% \pm 1.5\%$	$16.1\% \pm 1.3\%$	$6.1\% \pm 0.6\%$
GCN-VAE_IAF	$75.1\% \pm 0.8\%$	$59.2\% \pm 2.7\%$	$9.8\% \pm 0.8\%$	$5.8\% \pm 0.7\%$

2 CHOICE OF LOSS FUNCTION

The optimization function used for normalizing flow models in variational inference is commonly formulated as the free energy bound (Rezende and Mohamed, 2015):

$$\mathcal{F}(x) = \mathbb{E}_{q_0(z_0)}[\ln q_0(z_0)] - \mathbb{E}_{q_0(z_0)}[\log q(x, z_K)] - \mathbb{E}_{q_0(z_0)}\left[\sum_{k=1}^{K} \log \left|\det(J)\right|\right] \quad \text{(S1)}$$

where $\log|\det(J)|$ stand for the log-det-Jacobian term (Rezende and Mohamed, 2015) for the corresponding flow model. In our setup, this term would be added to the task-specific reconstruction loss. For our GCN-VAE_HouseholderSNF model, we compare using this loss function with the added Chamfer loss described in Eq. (2), against using the same loss function for our baseline VAE model as described in Eq. (1). All other model parameters are kept identical between the two trainings. Table S2 shows the results of this comparison with "Modified ELBO" signifying Eq. S1 with the added Chamfer term, and "Our Loss" signifying Eq. (1). It is evident that not modifying the loss for the VAE after adding the flow layers results in significantly better anomaly identification performance and as a result we utilize this strategy to train all other flow models presented in this study.

3 IMPACT OF NORMALIZING FLOWS ON THE LATENT SPACE PRIOR

To understand exactly how non-Gaussian the latent distributions become after passing through the normalizing flow layers, we attempt to visualize our 15 dimensional latent space via two methods. First, we create 2D histograms from multiple randomly chosen pairs of dimensions. Fig. S1 shows one such

Table S2. Anomaly detection performance across all channels, combined based on median scores.

Model	AUC	$\epsilon_S(\epsilon_B = 10^{-2})$	$\epsilon_S(\epsilon_B = 10^{-3})$	$\epsilon_S(\epsilon_B = 10^{-4})$
Modified ELBO	78.3%	57.1%	16.1%	6.6%
**Our Loss**	**86.4%**	**71.6%**	**34.0%**	**8.2%**

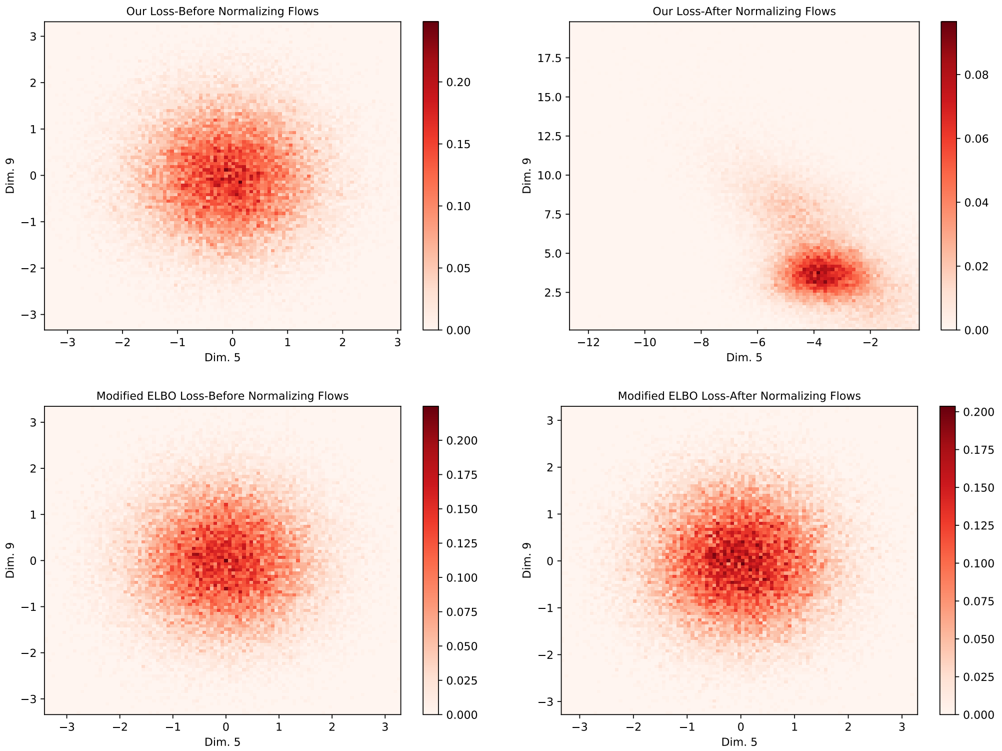

Figure S1. Latent space visualization by making histograms across arbitrarily chosen dimensions 5 and 9, before (left) and after normalizing flow transformations (right) with our loss function (top) and the modified ELBO loss (bottom).

distribution between dimensions 5 and 9. We also make an approximate visualisation by first performing a principal component analysis (PCA) to express the latent space in 2 dimensions, and then plotting the resulting 2D histogram as shown in Fig. S2.

We also show a comparison of the latent space obtained from the trainings with the two different loss functions, as described in the previous section of supplementary material. We see that our loss function results in a more complex, non-Gaussian distribution compared to the modified ELBO loss, and this is desirable to improve anomaly detection performance using VAEs. It is important to note that using our loss function may not necessarily correspond to better reconstruction of the input for the trained class (background samples) or better generation, but it rather contributes to a larger separation between the trained class and the non-trained class (signal samples) by making it harder for the decoder to reconstruct signal samples. As a result the anomaly identification performance increases regardless of whether the reconstruction of the background samples improves or not.

REFERENCES

Rezende, D. and Mohamed, S. (2015). Variational inference with normalizing flows. In *Proceedings of the 32nd International Conference on Machine Learning*, eds. F. Bach and D. Blei (Lille, France: PMLR),

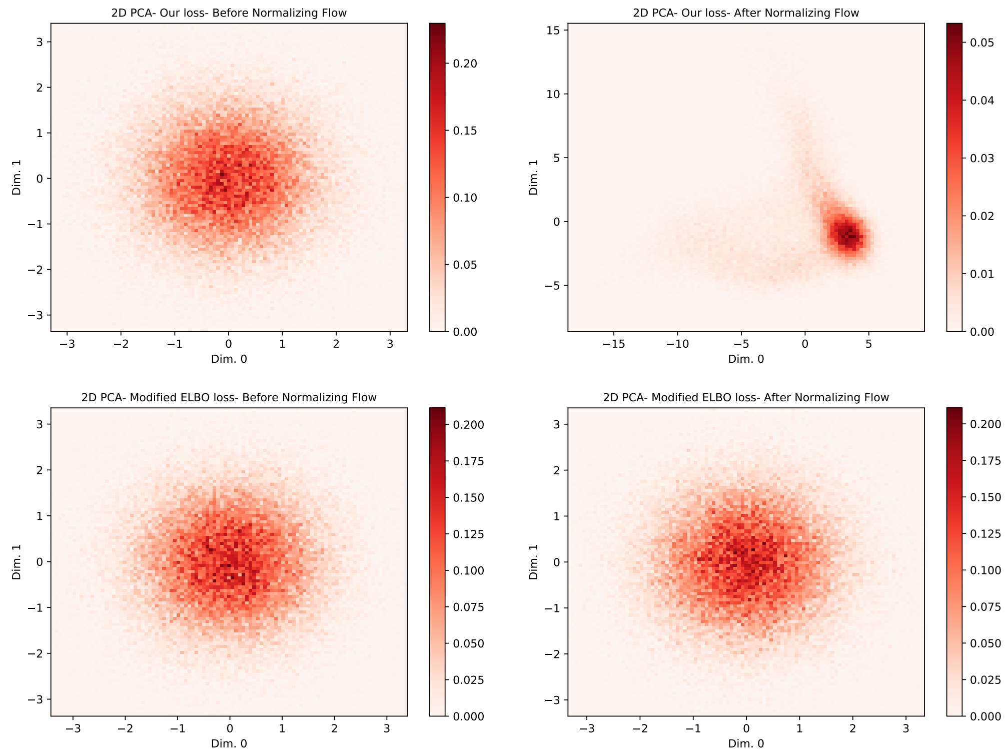

Figure S2. Latent space visualization after 2D PCA, before (left) and after normalizing flow transformations (right) with our loss function (top) and the modified ELBO loss (bottom).

vol. 37, 1530
